# A Logic-Based Psychotherapy Approach to Treating Patients Which Focuses on Faultless Logical Functioning: A Case Study Method

**DOI:** 10.3389/fpsyg.2017.02249

**Published:** 2017-12-22

**Authors:** Fernando Almeida, Diana Moreira

**Affiliations:** ^1^Department of Social and Behavioral Sciences, University Institute of Maia, Maia, Portugal; ^2^Abel Salazar Institute of Biomedical Sciences, University of Porto, Porto, Portugal; ^3^Laboratory of Neuropsychophysiology, Faculty of Psychology and Educational Sciences, University of Porto, Porto, Portugal; ^4^Portucalense Institute of Neuropsychology and Cognitive and Behavioral Neurosciences, University of Porto, Porto, Portugal

**Keywords:** logic-based psychotherapy, logical reasoning/functioning, interpersonal relationship, cognitive bias, behavior modification, case study

## Abstract

Many clinical patients present to mental health clinics with depressive symptoms, anxiety, psychosomatic complaints, and sleeping problems. These symptoms which originated may originate from marital problems, conflictual interpersonal relationships, problems in securing work, and housing issues, among many others. These issues might interfere which underlie the difficulties that with the ability of the patients face in maintaining faultless logical reasoning (FLR) and faultless logical functioning (FLF). FLR implies to assess correctly premises, rules, and conclusions. And FLF implies assessing not only FLR, but also the circumstances, life experience, personality, events that validate a conclusion. Almost always, the symptomatology is accompanied by intense emotional changes. Clinical experience shows that a logic-based psychotherapy (LBP) approach is not practiced, and that therapists’ resort to psychopharmacotherapy or other types of psychotherapeutic approaches that are not focused on logical reasoning and, especially, logical functioning. Because of this, patients do not learn to overcome their reasoning and functioning errors. The aim of this work was to investigate how LBP works to improve the patients’ ability to think and function in a faultless logical way. This work describes the case studies of three patients. For this purpose we described the treatment of three patients. With this psychotherapeutic approach, patients gain knowledge that can then be applied not only to the issues that led them to the consultation, but also to other problems they have experienced, thus creating a learning experience and helping to prevent such patients from becoming involved in similar problematic situations. This highlights that LBP is a way of treating symptoms that interfere on some level with daily functioning. This psychotherapeutic approach is relevant for improving patients’ quality of life, and it fills a gap in the literature by describing original case analyses.

## Introduction

Many patients approach us seeking relief from symptoms such as psychosomatic complaints, depression, anxiety, and sleeping problems. These symptoms are often accompanied by emotional disturbances and are related to life events such as marital problems, employment, financial problems, and conflicted interpersonal relationships (Almeida et al., 2016). Our clinical experience, as well as past research, informs us that many of these patients benefit more from the use of a logic-based psychotherapy (LBP) approach—either alone or combined with psychopharmacological therapy—than from pharmacological treatment alone. Accordingly, many people have difficulties in maintaining a faultless logical reasoning (FLR), and even if FLR is present, they have difficulties in maintaining faultless logical functioning (FLF).

Faultless logical reasoning implies to assess correctly premises, rules, and conclusions. And FLF implies assessing not only the premises, the rules, and the conclusions, but also the circumstances, life experience, personality, events that validate a conclusion. We define the LBP approach as the practice of studying patients to understand whether they function with faultless logic, particularly regarding the issues that underlie the origins (Cohen, 1987) of their symptomatology. In doing this, one also tries to understand whether the patient has lapses in logical reasoning and functioning within a broader perspective of their life. While similar approaches exist [e.g., rational-emotive behavior therapy (REBT); Ellis, 1995; logic-based therapy (LBT); Cohen, 2013, 2016], no other logic-based approach, to our knowledge, takes into account these individual differences and broader life perspectives. In its operational definition, LBP does not rely solely on the analysis of pure logic itself, but also entails analysis of the premises, rules, and conclusions (FLR), as well as the life experience, personality, circumstances, and events that validate such conclusions (FLF). Thus, it is essential to: (a) begin from premises that are accurate and adequately take into account the individual’s knowledge and associate other data that may or may not be present (this point is very important because patients frequently cite other reasons that have led to their current situation); (b) maintain FLR; (c) reach logically valid and faultless conclusions, for which the quality of interpretation is crucial; (d) select the correct conclusion; and (e) request information, in the event of not being able to select the correct conclusion (Almeida et al., 2015).

Individuals with a greater degree of FLF are less likely to experience depressive and anxious symptoms and are more likely to be functioning satisfactorily (Almeida et al., 2016). Difficulties in FLR and FLF lead to misunderstandings, conflicts, inconsequential realizations, ruptures between people, and inappropriate boycotts toward other people’s, or an organization’s, work (Almeida et al., 2015). These difficulties are often serious and accompany the individual throughout life, and it is absurd not to take advantage of the clinical contacts of patients to help them attain improved logical reasoning and logical functioning (Almeida et al., 2016).

Psychotherapeutic approaches such as REBT (Ellis, 1995), LBT (Cohen, 2013, 2016), cognitive behavioral therapy (CBT; e.g., Stewart-Sicking, 2015), narrative psychotherapy (e.g., Boritz et al., 2014), and interpersonal psychotherapy (e.g., O’Shea et al., 2015) can focus on FLR and FLF as a consequence of therapy, but these alternative therapies do not explicitly center on analyzing and treating FLR and FLF, especially within the context of interpersonal relationships and broader life contexts. For example, in LBP, analysis focuses on what occurs with logical reasoning and functioning regardless of whether or not the patient is displaying a pathological emotional state. LBP is relevant even when individuals are, apparently, thinking correctly, since LBP focuses not only on the way individuals think, but also on the way they function (Almeida et al., 2016). In comparison, CBT focuses on correcting errors of thought without the main focus on current experiences and interpersonal relationships, including type of communication and, fundamentally, the words specifically verbalized by the parties (and not only prosody, but also pitch, mode, intensity, and frequency) (e.g., Cristea et al., 2013; Goldman et al., 2013). Narrative psychotherapy (NP) is distinctly different from LBP because the narrative does not always correspond to what actually happened in detail, and even if NP seeks to remake logical reasoning, it is not concerned with exploring logical functioning and does not provide the best possible way to analyze the situation (e.g., Boritz et al., 2011; Androutsopoulou, 2013). Finally, interpersonal psychotherapy looks to solve psychiatric symptoms rather than modify personal psychological structures or personality characteristics (e.g., Binder and Betan, 2013; Bernecker et al., 2014), while LBP distinctly considers focus its object of analysis on the logical reasoning and functioning with which patients became involved in the conflict (Almeida et al., 2016).

Mahoney has formerly implied that the theories of both Beck and Ellis are rationalistic (Mahoney and Gabriel, 1987). However, Wessler argued the opposite—that both are, in theory, constructivists, citing their common appeal to the previous quote from Epictetus: “who might be called the patron philosopher of constructivists” (Wessler, 1992, p. 622). Nonetheless, cognitive-behavioral psychotherapy seeks to identify errors in thinking and cognitive distortions, among other cognitive lapses (e.g., maximization) displayed by the patient, in order to guide the patient toward appropriate cognitive strategies.

A LBP approach is the only psychotherapy approach that possesses the refinement, accuracy, amplitude, and depth needed to study FLR and FLF. The other aforesaid psychotherapies center on what occurs within the individual in a context of mental psychopathology, whereas LBP can be applied regardless of whether or not the patient is displaying a pathological emotional state. LBP is heavily focused not only on what happens inside the individual, but also on what occurs in relationships and on the way in which the individual evaluates the behavior and personality of others (Almeida et al., 2016). To function appropriately, the individual has to be able to evaluate the countless alternatives available to him or her and choose the one that is most suited to every moment, person, and situation. LBP utilizes a more systematic and thorough technique than any other form of psychotherapy.

The work on FLF and FLR is an approach that can be applied to any type of psychotherapy, and even in any consultation not necessarily involved in a context of psychotherapy. Focusing on FLR and FLF requires: (a) the therapist to show absolute empathy toward the patient; (b) trust in the psychotherapist; (c) a psychotherapist with a working knowledge of FLR and FLF, otherwise the psychotherapist may be “caught out” and thereby no longer held in high regard by the patient; (d) the ability of the patient to carry out the necessary rational work; (e) availability of the patient to discuss the issue from a more logical and less emotional perspective; and (f) the patient’s readiness to learn (e.g., Almeida et al., 2016).

Psychotherapists may indeed explore the patient’s reasoning, as well as the emotions that result from it. However, if psychotherapists fail to explore FLF—namely, when patients think with apparent FLR—patients will not understand the source of their inaccurate or inadequate thought processes and/or functioning. Therefore, by not evaluating the degree of FLF, psychotherapists hinder more effective intervention, even if they manage to accurately assess the level of FLR (Almeida et al., 2016). LBP is relevant for a broader range of situations, and in particular, all situations that are underpinned by conflicts.

By recognizing that each individual is unique, case studies provide relevant evidence to support the application of psychological therapies (Allport, 1962). This methodology entails several intrinsic advantages, such as internal validity and real-life application in clinical settings.

In the context of previous research, the present study manages to fill existing gaps in the literature using original case analyses, thus making this original research. The evidence shows that LBP is effective yet under-recognized by research and under-used by therapists (Almeida et al., 2016). In the following sections, we outline three case examples of how LBP has been used to treat patients, and discuss the potential long-term implications of adopting an LBP approach.

### Case Introductions

The psychotherapeutic approach “logic-based psychotherapy” was implemented in clinical practice in Porto (in the north of Portugal) and focused on patients that exhibited depressive and anxious symptoms, including psychosomatic complaints, sleeping problems, conjugal/family conflicts and other relational conflicts, and employment and financial problems, among others. Once the local ethics committee (Committee of Clínica Capitólio) had approved the study, the patients were notified of the purpose of the study and invited to voluntarily participate in the study after signing an informed consent form.

### Objectives

This psychotherapeutic approach was adopted because, when collecting the clinical history, we examined the logic the patients had used to analyze situations, and particularly, how they had functioned in the situations that had initiated the conflicts resulting in their symptomatology.

The specific objectives were (1) to improve the patients’ ability to think and function in a faultless logical way, (2) to restore lost relationships, and (3) to prevent future conflicts.

The planned technical tasks consisted of:

(1) obtaining a detailed clinical history with the patient, and if allowed by the patient, the clinical history would be complemented by hearing in another session a family member with whom he/she lives, and in the presence of the patient;
(2) examining the causes of the conflict with the patient;
(3) exploring how the patient analyzed the different issues, interests, and behaviors of those who were involved, including him- or herself;
(4) seeking to gain a thorough understanding of the issues, i.e., beyond what the patient explains spontaneously;
(5) making sure that the patient intends, and is able, to analyze his/her behavior;
(6) conducting an analysis with the patient about his/her behavior and presenting possible alternatives to what he/she thought or did;
(7) helping the patients understand the procedural errors they had committed and analyzing whether they had experienced any other lapses in thinking or logical functioning in their previous history;
(8) displaying the capacity to analyze potentially conflictual situations with the patient and helping him/her to analyze them too.

The hypotheses were (1) with LBP the patients’ ability to think and function in a faultless logical way will improve, (2) LBP will increase probability to restore lost relationships, and (3) LBP will teach patients to prevent conflicts.

The cases described below were selected because they were paradigmatic of the usefulness of this psychotherapeutic approach—LBP.

## Materials and Methods

### Participants

The sample consisted of three participants residing within a radius of 120 km from the city of Porto (Portugal), who were treated using this psychotherapeutic approach.

The inclusion criteria comprised being of Portuguese nationality, and having exhibited depressive symptoms, anxiety, psychosomatic complaints, or sleeping problems. Participants with a nationality other than Portuguese were excluded (because of language problems), as well as participants without sufficient intelligence (clinical evaluation) to address the issue with an enhanced degree of sophistication. Diagnosis was made in accordance with the Diagnostic and Statistical Manual of Mental Disorders (DSM-IV-TR; American Psychiatric Association, 2002). LBP has been implemented between 1990 and 2016. The three cases selected demonstrated the therapeutic importance of this psychotherapeutic approach, and their diagnoses were not affected by revisions in the DSM criteria.

### Data Collection Methods

#### Socio-demographic Data

The socio-demographic data and patients’ characteristics were obtained by interview (e.g., name, age, sex, marital status, birthplace, education, occupation, employment).

#### Clinical Data

(1) By means of clinical interviews, data was gathered from the patient and other family members (e.g., the presenting complaint, personal and familial background, history of this and other complaints, living situation, social support, previous use of psychology/psychiatry, medication, and current coping strategies). (2) The causes of the conflict were examined with the patient, along with the way he/she thought, how the other(s) answered, how he/she reacted, and what the patient did; in addition, all other possibilities that appeared logical to the patient, to the other person(s) involved in the conflict, and to the other family members were investigated.

### Applying the Logic-Based Psychotherapy Approach: Case 1

To better understand what is being discussed, we will describe the case of a 50-year-old man, who presented with nervousness, irritability, daily insomnia, concentration problems, and psychosomatic complaints (e.g., palpitations, the sensation of having a lump in his throat). JP (not his actual initials) was able to work, although he reported a reduced quality of working life, and he was experiencing increased levels of social isolation, during the last 8 months.

As part of the assessment process, JP completed a life timeline with the psychotherapist. JP recognized his own strengths and the skills he had used to independently overcome a period of generalized anxiety in the past.

Of all the psychosomatic complaints, the complaint that most disturbed JP was insomnia. The patient essentially wanted us to medicate him to resolve his insomnia and other psychosomatic complaints, with no attempt to discuss the possible reasons that may have contributed to the onset of his complaints.

However, before eventually medicating the patient, we tried to dissect his life story with him. We then found that the onset of the complaints overlapped in time with a conflict the patient had had with a coworker. The latter had opened a sealed letter that the patient had asked him to deliver to the Post Office, and had added the patient’s document to other documents from the company, eventually sending all the documents, in the same envelope, to the Post Office. This had led the patient to have a heated argument with his colleague, which had in turn affected the atmosphere at work, necessitating the intervention of a superior on more than one occasion.

In fact, the company did indeed have a policy of inserting any documents being sent to the same address into a single envelope, for economical purposes. However, as we stated previously, the patient had specifically requested that his correspondence be sent in the envelope he had handed to his colleague, and his indignation had resulted from his colleague violating that request. So, it appeared that the patient had a reason to be angry. We could have focused our work with the patient on analyzing the emotions he had felt, on the way he had responded, and on the inadequate discrepancy of having expressed himself that way at his workplace. Nevertheless, our primary goal was to analyze the patient’s level of FLR, and particularly his level of FLF, without affecting any subsequent analysis of his emotions. Our concern was always to consider whether the patient had acted with faultless logic, for which we needed to gather all the relevant information.

We regard the sessions as a unique moment of teaching/learning, as a moment in which we can help the patient reason with better logic, in cases where, as so often happens, the patient has expressed lapses in logic.

In JP’s case, we sought to immediately dissect the situation and we discovered that, when the patient had handed the envelope to his colleague to put in the mail, he did not offer any amount of money to pay for a postage stamp. Thus, the colleague treated the correspondence as if it were from the company and not a private correspondence.

When we confronted the patient with his misstep, he was perplexed; that oversight had never occurred to him, much less that it could have legitimated his colleague’s behavior. But, when confronted by us, he admitted that his behavior had led his colleague to treat his correspondence as if it were the company’s. The patient’s anger quickly diminished. Although he continued to argue that his colleague could have asked for the money for the postage stamp, he realized that he was partially to blame for the outcome.

JP was diagnosed as suffering from Generalized Anxiety Disorder and he was medicated with an anxiolytic. Additionally, the patient was offered 10 1-h psychotherapy sessions over the course of the next 12 months, including the assessment sessions. The intervention sessions aimed to analyze with the patient the conflictual situations he had found himself in—namely, the one that may have led to actual complaints, and two other similar situations in which he had precipitated serious conflict: one in another company that had led to his dismissal, and another with a family member, in which he had lacked breadth of evaluation. In these sessions he learned to practice faultless logic.

### Applying the Logic-Based Psychotherapy Approach: Case 2

AG, aged 45, who was a hospital doctor, was able to work, and did not report serious restrictions in work activities due to the symptoms he was experiencing.

As part of the assessment process, AG completed a life timeline with the psychotherapist. As in the procedure conducted with JP, AG recognized his own strengths and the skills he had used to independently overcome a period of generalized anxiety in the past, when he had had a dispute with his brother.

AG was found to be depressed, angry, anxious, irritated, and with sleep disorders. He expressed concerns about his work and exhibited concentration problems. He was a very distinguished and reputable professional. He had completed an internship in one of the world’s leading centers for his specialty and had a particular interest in research. However, the director of the institution where he worked wanted him to work only as a clinician and not as a researcher. The director had not allowed him to start a laboratory research, because he claimed that the research would be very expensive, and besides, the hospital had hired him to be a doctor and not a researcher. AG agreed that he had been hired as a clinician, but nevertheless insisted on starting the research. When this was refused, he initiated a lengthy confrontation, in which he caused a bad atmosphere within both the service and the institution. He ridiculed the director of the institution, labeling him as—among other inculpations—incompetent, mediocre, and a person “without a vision.” As a consequence, the director started a disciplinary process against him.

In the first session, we medicated the patient with a serotoninergic antidepressive for his Mixed Anxiety and Depression Disorder, and although we wanted to analyze the issue with him, we refrained from doing so. This was because we had not yet established a relationship of sufficient trust, nor did we feel that the patient was prepared to discuss the conflict with us, which would in fact come about in the third session. In the end of this session, we suggested the patient to think about what he would do if he was the director of the hospital. In the fourth session the patient agreed that he had not operated with FLF and that he had let himself be carried away by his emotions and by what he thought was best for him and for the institution. Additionally, he wanted to discuss with us another aspect of his personal life that had troubled him because it entailed the severing of his relationship with a brother, due to an issue focused around property shares. In this conflict, neither the patient nor his brother—both well-endowed with intelligence—had utilized FLR or FLF in their dealings with one another. In this case with the brother, if we did not evaluate the words, pitch, mode, and intensity specifically verbalized by the protagonists, and other rationale options, we would be convinced that the rationale option of the patient was adequate.

AG was offered four 1-h psychotherapy sessions over the course of 4 months, including the assessment sessions. He was very intelligent, learned quickly, and maintained a very interesting career.

### Applying the Logic-Based Psychotherapy Approach: Case 3

The patient, MB, aged 52, was a married man (his wife was a homemaker) with two children at university, with a leadership position in a company. *He agreed to continue working for this company after it had relocated 70 km away from his home.* Moreover, *he was promised a sum of money that, 9 months after the workplace transfer, he had yet to receive*. This non-compliance led to an enormous unease between the patient and the company administrator, with a consequent exacerbating conflict.

When the patient came to us, he was seething with hatred and anger, he could not sleep, he had depressed mood, feelings of hopelessness, and several psychosomatic complaints. We medicated him with an antidepressive and a hypnotic (in SOS) for Adjustment Disorder with Depressed Mood and dissected the reasons for the conflict in the first, second, and third sessions.

Apparently, the patient had the right to feel angry. However, examining the problem allowed us to understand that the company was going through a period of great turbulence, and there were dozens more workers whose promises—made at the time of the transfer—remained unfulfilled. The patient had talked to the owner of the company about his concerns, and the latter sent him to speak with the administrator. *Although the administrator had never told the patient he would not pay him, he was indeed dragging out the situation. The patient argued that it was the administrator who did not want to pay him his due, not his boss, particularly given the fact that the latter had always proved to be a good friend to him*. It was undisputed that the patient was a reputed employee, whom they held in high regard and whose monthly wage was well above what he would be entitled to were he to become unemployed (the patient only had a few academic qualifications and it was doubtful that he would earn an equivalent wage working for another company).

When the patient came to us, he had consulted another professional who had agreed with his demanding confrontational stance. Together with the patient we outlined the following logical reasoning: (a) the patient’s boss was very likely aware of the patient’s demands; (b) the boss had sent the patient to the administrator, to whom he had granted full powers to manage the company; (c) the administrator did not fulfill the patient’s demands and other similar employees’ demands: (d) it seemed to us that the decision did not rest solely with the administrator, but also involved the boss; (e) the issue of the non-payment of the promised sum was not a personal issue of the administrator (as the patient thought), but of the company, and even, very likely, by order of the boss himself.

This change of perspective caused in the patient a change in outlook and in the quantity and quality of anger that he felt. His anger diminished because his focus no longer rested solely on the individual he regarded as hostile (the administrator), but became more directed toward the individual he had held in high esteem (his boss) and the company.

It would have made sense for the boss and the administrator of the company to properly explain the reasons why they had not yet proceeded as promised. The truth is, however, they had not. Nonetheless, during treatment, the patient was able to understand that the non-payment of what was due to him did not result from a personal animosity on behalf of the administrator, but because of a decision made, almost certainly, between the boss and the administrator. This allowed us to extend the scope of the analysis, thus: (a) the company was undergoing a period of turbulence, but the promised payment had not been formally refused; (b) the company was about to lay off dozens of workers, but the patient had been invited to continue working in the company; (c) the wage the patient earned was twice, or close to that, what he would earn in another company, and yet no corresponding drop in salary had ever been proposed; (d) if the company had fulfilled the payment agreed with him, it would have had to do the same for the other workers, and the company was not in a position to do so.

Having arrived at this point, we highlighted to the patient that he had two equitable ways to assert his rights: (a) the one he had adopted, and which he had a right to, given that commitments are made to be fulfilled, and (b) an expectant attitude in which he took into consideration the company’s position. After a more meticulous analysis of the company’s position, the patient decided to adopt the latter stance, an attitude which, indeed, seems more aligned with logical thinking. We saw this patient for about a year, during which time he greatly improved not only his clinical condition, but also his level of FLR and FLF. He continued to work for the same company, which was experiencing a little more stability than previously and, furthermore, had paid him all that he had been promised.

In italics are presented vignettes when the patient provides reasoning that is not faultless, to better show how LBP was actually performed.

## Results

By applying this approach, patients’ clinical evolution showed a notorious improvement: they became more comfortable with themselves and with their environment, including interpersonal relationships. They experienced greater inner peace and displayed a calmer attitude than the aggressive stance that had led to the aforementioned symptomatology.

The newfound clarity and distention of judgment observed in these patients were so sudden, it was as if a light had been shone directly onto them.

All three patients had enjoyed the LBP approach—namely, the ability to accurately think and function with faultless logic. They understood and learned to think and function within all the parameters involved in the situations and improved their capacity to think about the best choice to select. They also understood how emotions can sometimes disturb logic and precipitate an erroneous behavior if the logic is not faultless.

The most noteworthy finding was that, once patients have learned this form of thinking and functioning, they no longer dismiss it.

## Discussion

Many patients come to us with underlying mental disorders to which there are interpersonal conflicts related to life events such as marital problems, employment, and financial problems (Almeida et al., 2016). As we have been able to exemplify with the clinical cases discussed, psychotherapists are often faced with patients who display symptoms associated with problems caused by the patient’s difficulties in terms of FLR and FLF. Almost always, this symptomatology (depression, anxiety, somatic complaints) is accompanied by intense emotional changes.

Research on emotion in psychotherapy demonstrates the importance of emotion in human functioning and psychotherapeutic change (e.g., Greenberg, 2008). Accessing and exploring emotions, within the context of a secure therapeutic relationship, leads to therapeutic changes which have been widely held by several psychotherapeutic theories (e.g., Rogers, 1951; Perls, 1969; Kohut, 1977; Bowlby, 1980). There is increasing evidence on the importance of emotion knowledge in enhancing social competence and healthy development (Mayer and Salovey, 1997). Emotions are complicated, and have complex interactions with other psychological states (Ong et al., 2015). As we have said, LBP has impact in emotion because many patients without FLR and FLF experience emotional and problematic changes determined by their thoughts and acts. Think better result in more healthy emotions.

Similar to past research, the psychiatric and psychological consultation we conducted proved to be pivotal for the patients to analyze the difficulties they had encountered (Androutsopoulou, 2013; Cort et al., 2014; Kivlighan, 2014; Meffert et al., 2014; Renaud et al., 2014; Ryum et al., 2014; Sools and Schuhmann, 2014; Stewart-Sicking, 2015). Specifically in terms of FLR and FLF, the consultations helped patients analyze their difficulties, logic, emotions, and behaviors within the context of life situations, while we continued to work with them to improve on possible shortcomings they had demonstrated.

The specific objectives of LBP were to improve the patients’ ability to think and function in a faultless, logical way, to restore lost relationships, and to prevent future conflicts. With LBP, patients apply the knowledge they have gained not only to the problems that brought them to the consultation, but also to other problems they may experience, thus providing learning and helping to prevent similar problematic situations from developing in the future (Almeida et al., 2015). LBP does not require a lengthy intervention because it is based on the investigation of faulty FLRs that are mostly, if not always, on a conscious level, i.e., likely easily accessible to the patients. However, FLF is more difficult to demonstrate and can need the contribution of other person if possible and allowed by the patient. Another important aspect is that improving competence in terms of logical reasoning and functioning contributes to improved emotional processing. This is either because the individual learns to cognitively assess situations better, or because he/she is less likely to get involved in problematic situations, with both leading to a positive impact on the emotional component (Goldman et al., 2013). Experience tells us that when the patients become aware that their reasoning and functioning are not the most appropriate, the emotional component improves, invariably contributing, moreover, to them being more profound and competent, and less hasty in their analyses and in their behaviors.

Logic-based psychotherapy has the advantage of providing a tool that will accompany the individual throughout life, introducing a posture in which the individual learns to analyze him- or herself permanently and to think with FLR. This may correct insufficiencies at this level and assist in developing FLF, with very beneficial repercussions at both the interpersonal, intrapersonal, and social levels. LBP further assists patients to be more profound and rigorous in their analysis and their responses. Thus, they become more logical and learn to consider alternatives for their reasoning and functioning, and to improve their interpersonal relationships. Accordingly, LBP is very enriching for patients and people in general, and can be developed both as a therapeutic plan, and as an educational-level plan, in circumstances not necessarily linked to psychotherapy (Almeida et al., 2016).

The work carried out with these patients supports the notion that many patients do not have a faultless FLR/FLF, and that LBP can be considered as a valuable treatment. Intelligent patients learn very quickly and become captivated by the enjoyment of learning to think with FLR and proceed with FLF. Some of them may teach other family members to think with FLR. The added value that this brings to their lives is very relevant, because it may apply not only to the situations originating the psychopathology, but also to other contexts within the individual’s life. So, when patients look to us, to provide LBP can be a very important moment of treatment, learning, and change. Nonetheless, it should be emphasized that the changes associated with it have not yet been systematically measured. We believe that these changes are indeed measurable, though we never did this with our patients. This will be the next step in our approach. To our knowledge, this kind of approach has not yet been studied. Assessment by means of LBP involves a very meticulous investigation that is not only about the way the patients think, but also how they function—namely, in their interpersonal relationship and the choices made by them. We believe that no other psychotherapy technique exists whereby the study of logical reasoning and functioning is performed so meticulously and profoundly as in LBP—namely, when patients think with apparent FLR. Learning with FLR and FLF provides skills and originates behaviors that can be analyzed and measured. In this context, tests relating to FLF and FLR can be created and validated to determine to extent to which an improvement in thinking and logical operation positively influences the patients’ symptoms and behaviors.

This type of approach will become mandatory in psychotherapy. This is because it makes no sense to not have the potential to thoroughly investigate how someone acts and works, and learns to study all possible alternatives, as well as to teach people how to think and act better (Stangier et al., 2010; Cristea et al., 2013; Goldman et al., 2013; Rodríguez-Moya and Fernández-Belinchón, 2013; Renaud et al., 2014; Ryum et al., 2014; Stewart-Sicking, 2015). As with psychotherapy, learning FLR/FLF makes sense in organizations such as schools and other settings, especially, because this learning can be reflected on for long periods of time throughout the entire course of an individual’s life.

### Limitations of This Study

Further research on this issue needs to be conducted in the future. The ongoing study of a few cases is more relevant in the study of a new psychotherapeutic approach than even an open systematic analysis of a larger group or randomized controlled trials. Although, the description of a few case reports can be of relevance only after a systematic analysis with standardized tools of a larger group of patients undergoing LBP has been conducted and reported. Certain qualities are required of the patients for this technique to be successfully applied, such as intelligence levels that allow them to examine their thoughts and how they functioned within a specific context, an open attitude to facilitate correct analysis of the situation, and confidence in the psychotherapist, as well as the capacity to face what they find uncomfortable. LBP is fairly new and needs to be established further both theoretically and methodologically. Moreover, this approach needs to be replicated and tested for validity and reliability to support its utility and usefulness. LBP is not very different from behavioral chain analysis. However, LBP is a more ongoing bilateral excursion of patient and therapist, namely in the study of FLF.

There are various reasons why FLR and FLF may not be considered. Some are inherent to the psychotherapists themselves, while others are inherent to the patients, to the intricacy or sensitivity of the situation to be approached, and when this approach should be initiated (Almeida et al., 2016). The therapeutic parameters required for the provision of LBP are, in essence, not different from those required in any psychotherapy.

The reasons inherent to the patient may also be multiple: (a) the patient does not have (temporarily or permanently) the mental and intellectual availability—mainly due to affective reasons—to allow the issue to be approached from this perspective; (b) the patient and psychotherapist do not manage to establish a relationship that fosters the openness and trust necessary to engage in this type of approach; (c) the patient lacks the ability to self-analyze, and so is unable to recognize any “faulty logic”; (d) the patient does not have sufficient intelligence to address the issue with an enhanced degree of sophistication; (e) the patient is conditioned/limited by prejudice, religious, and political convictions, along with other beliefs, that do not serve to facilitate analysis of the issue from aforementioned perspective.

Owing to the complexity of the issue such an approach may be ill advised because: (a) the event led to a loss; (b) the event led to an altered mindset; (c) there was insufficient time to allow the patient to be able to logically analyze the event/situation with the psychotherapist; (d) a rational analysis of the event/situation may lead to more severely heightened negative emotions in the patient than would be the case if no analysis were conducted; and (e) any benefit afforded to the patient as a result of a rational analysis of the event/situation may be of questionable value.

## Ethics Statement

This study was carried out in accordance with the recommendations of American Psychological Association with written informed consent from all subjects. All subjects gave written informed consent in accordance with the Declaration of Helsinki. The protocol was approved by the local committee.

## Author Contributions

FA designed the study. He also conducted literature searches and provided summaries of previous research studies. DM and FA wrote the manuscript. DM contributed to and have approved the final manuscript.

## Conflict of Interest Statement

The authors declare that the research was conducted in the absence of any commercial or financial relationships that could be construed as a potential conflict of interest.
